# Corylin Inhibits Vascular Cell Inflammation, Proliferation and Migration and Reduces Atherosclerosis in ApoE-Deficient Mice

**DOI:** 10.3390/antiox9040275

**Published:** 2020-03-25

**Authors:** Chin-Chuan Chen, Hung-Yuan Li, Yann-Lii Leu, Yu-Ju Chen, Chia-Jen Wang, Shu-Huei Wang

**Affiliations:** 1 Graduate Institute of Natural Products, Chang Gung University, Taoyuan 33302, Taiwan; chinchuan@mail.cgu.edu.tw (C.-C.C.); ylleu@mail.cgu.edu.tw (Y.-L.L.); 101712103@gms.tcu.edu.tw (Y.-J.C.); 2 Chinese Herbal Medicine Research Team, Healthy Aging Research Center, Chang Gung University, Taoyuan 33302, Taiwan; 3 Tissue Bank, Chang Gung Memorial Hospital, Taoyuan 33305, Taiwan; 4 Department of Internal Medicine, National Taiwan University Hospital, Taipei 10002, Taiwan; larsli@ntuh.gov.tw; 5 Center for Traditional Chinese Medicine, Chang Gung Memorial Hospital, Taoyuan 33305, Taiwan; 6 Cell Therapy Center, Chang Gung Memorial Hospital, Taoyuan 33378, Taiwan; wangcj225@cgmh.org.tw; 7 Department of Anatomy and Cell Biology, College of Medicine, National Taiwan University, Taipei 10051, Taiwan

**Keywords:** atherosclerosis, corylin, ROS, inflammation, mitofission

## Abstract

Atherosclerosis is a complex disease that includes several events, including reactive oxygen species (ROS) stress, inflammation, endothelial dysfunction, lipid deposition, and vascular smooth muscle cell (VSMC) proliferation and migration, which result in atherosclerotic plaque formation. Corylin, a flavonoid compound, is known to exhibit antioxidative, anti-inflammatory and antiproliferative effects. However, it remains unknown whether corylin could modulate atherogenesis. Here, we identified the anti-inflammatory effect of corylin in tumor necrosis factor-α (TNF-α)-induced vascular cells. In human umbilical vein endothelial cells (HUVECs), corylin suppressed TNF-α-induced monocyte adhesion to the HUVECs and transmigration by downregulating the ROS/JNK/nuclear factor-kappa beta (NF-κB) p65 pathway. In VSMCs, corylin inhibited TNF-α-induced monocyte adhesion by suppressing ROS production, mitogen-activated protein kinase (MAPK) phosphorylation and NF-κB p65 translocation. In platelet-derived growth factor-BB (PDGF-BB)-induced VSMCs, corylin inhibited PDGF-BB-induced VSMC proliferation and migration through regulating the mammalian target of rapamycin (mTOR)/dynamin-1-like protein 1 (Drp1) signaling cascade. In addition, corylin treatment not only attenuated atherosclerotic lesions, ROS production, vascular cell adhesion protein-1 (VCAM-1) expression, monocyte adhesion and VSMC proliferation in apolipoprotein E (ApoE)-deficient mice but also inhibited neointimal hyperplasia in endothelial-denuded mice. Thus, corylin may be a potential prevention and treatment for atherosclerosis.

## 1. Introduction

Atherosclerosis causes the majority of deaths worldwide, and atherosclerosis progression is characterized by chronic inflammation, reactive oxygen species (ROS) generation, endothelial dysfunction, abnormal vascular smooth muscle cell (VSMC) proliferation and migration and plaque formation [1]. Proinflammatory cytokines and excessive ROS production cause endothelial cell activation and trigger monocyte adherence by upregulating cell adhesion molecules, such as vascular cell adhesion protein-1 (VCAM-1). As the disease progresses, the accumulation and interaction of transmigrated leukocytes with VSMCs increases the production of cytokines or growth factors, such as platelet-derived growth factor-BB (PDGF-BB), and then facilitates VSMC proliferation and migration, contributing to further atherosclerosis exacerbation [2]. Thus, potent new anti-inflammatory, antiproliferative and antimigratory agents for preventing atherosclerosis are urgently needed.

*Psoralea corylifolia* L. (Fabaceae) is among the most popular traditional Chinese medicines and has been shown to have antimicrobial activity [3], anticancer effects [4], and antioxidant activity [5,6] and to prevent diabetes [7], protect against palmitate-induced neuronal apoptosis [8], and inhibit high-fat diet-induced hepatic disease [9]. In particular, corylin, a flavonoid compound extracted from *Psoralea corylifolia* L., has been shown to stimulate osteoblast proliferation [10], attenuate lipopolysaccharide (LPS)- or interleukin-6 (IL-6)-induced inflammatory responses [11,12], suppress hepatocellular carcinoma progression [13], ameliorates hyperlipidemia, insulin resistance and atherosclerosis [14] and increase hepatocellular carcinoma cell sensitivity to chemotherapy and radiotherapy [15]. These protective effects appear critical in preventing the development of atherosclerosis. Although inflammation, oxidation, proliferation, and migration of endothelial cells and VSMCs play important roles in atherosclerosis progression, the anti-inflammatory, anti-oxidative, antimigratory and antiproliferative effects of corylin on endothelial cells and VSMCs remain unknown. Thus, it is important to elucidate the function and regulation of corylin on atherosclerotic endothelial and VSMCs for the clinical therapeutic application of corylin.

The aim of the present study was therefore to investigate the effects and mechanisms of action of corylin on adhesion molecule accumulation in tumor necrosis factor-α (TNF-α)-treated human umbilical vein endothelial cells (HUVECs) and VSMCs and its antiproliferative and antimigratory effects in PDGF-BB-treated VSMCs. This study showed that corylin treatment dramatically decreased TNF-α-stimulated VCAM-1 expression and monocyte adherence in HUVECs and VSMCs through inhibiting ROS/mitogen-activated protein kinase (MAPK)/NF-κB p65 activation. In addition, corylin inhibited PDGF-BB-induced VSMC proliferation and migration through mammalian target of rapamycin (mTOR)/dynamin-1-like protein 1 (Drp1) regulation. Furthermore, the results showed that corylin dramatically lessened the atherosclerotic plaque in the apolipoprotein E (ApoE)-deficient mice fed a high-cholesterol diet and suppressed neointimal hyperplasia in denudated-femoral arteries in vivo. These data suggested that corylin could be applied as a therapeutic agent for atherosclerosis.

## 2. Materials and Methods

### 2.1. Materials and Reagents

Polyclonal rabbit IgG antibodies against human GAPDH, β-actin, phospho-/total P38, phospho-/total ERK1/2, phospho-/total JNK, phospho-/total Drp1 and phospho-/total NF-κB p65, Cyclin E, Cyclin D1, CDK2, CDK4, BrdU, NOX1, and NOX4 and horseradish peroxidase (HRP)-conjugated goat anti-rabbit IgG antibodies were purchased from GeneTex (Irvine, CA, USA). A rabbit IgG isotype control antibody was purchased from GeneTex. A monoclonal rabbit antibody against human VCAM-1 was purchased from Santa Cruz Biotechnology (Santa Cruz, CA, USA). PD98059 was purchased from LC Labs (Woburn, MA, USA). SB203580 and SP600125 were purchased from Selleck Chemicals (Houston, TX, USA). PDGF-BB and TNF-α were purchased from PeproTech (Co, Rocky Hill, NJ, USA). Dichloro-dihydro-fluorescein diacetate (DCFH-DA), Dihydroethidium (DHE), *N*-acetyl-l-cysteine (NAC), diphenyleneiodonium (DPI), mitochondrial division inhibitor 1 (mdivi-1) and TRIzol reagent were purchased from Sigma-Aldrich (St. Louis, MO, USA). BCECF-AM and MitoTracker were purchased from Molecular Probes (Invitrogen, Carlsbad, CA, USA). A FITC-conjugated goat anti-mouse IgG antibody was purchased from Jackson ImmunoResearch (West Grove, PA, USA). Corylin (purity > 95.3%) was purchased from Shanghai BS Bio-Tech Co., Ltd. (Shanghai, China).

### 2.2. Cell Culture

HUVECs were obtained from the Bioresource Collection and Research Center (BCRC) (Hsinchu, Taiwan) and grown at 37 °C in endothelial cell medium (Lonza, Walkersville, MD, USA) containing penicillin-streptomycin (1%) and endothelial cell growth supplement at 37 °C in an incubator containing 5% CO_2_. U937 cells were purchased from BCRC and grown in RPMI-1640 medium (Life Technologies; Carlsbad, CA, USA) supplemented with 10% fetal bovine serum (FBS) and 1% antibiotic/antimycotic solution at 37 °C in an incubator containing 5% CO_2_. A7r5 VSMC and RAW264.7 cells were purchased from BCRC and cultured in DMEM (Life Technologies; Carlsbad, CA, USA) containing 10% fetal bovine serum (FBS) and 1% antibiotic/antimycotic solution at 37 °C in an incubator containing 5% CO_2_. Before conducting our experiments, the VSMCs were serum-starved for 24 h.

### 2.3. Sulforhodamine B (SRB) Assay

Cells were treated with various concentrations of corylin or TNF-α for 24 h. Then, cell viability was determined using an SRB assay according to the manufacturer’s specifications.

### 2.4. RNA Preparation and Quantitative PCR

After cells treated with corylin and TNF-α, total RNA was extracted using TRIzol reagent. cDNA synthesis was using an RT kit (Yeastern Biotech Co., Ltd, Taipei, Taiwan). The real-time quantitative PCR was performed by using SYBR Green PCR premix (Thermo, Wilmington, NC, USA). LightCycler 480 (Roche, Germany) was used for analysis. The β-actin gene was used for normalization. The primer sequences were VCAM-1(170210H7): 5′-ATCTTCTGCTCGGCAAGTC-3′ (forward) and 5′-GTTCTGACCTACATCTGGAGTG-3′ (reverse).

### 2.5. Western Blot Analysis

Treated cell and tissue lysates were subjected to sodium dodecyl sulfate polyacrylamide gel electrophoresis (SDS-PAGE) and then transferred to PVDF membranes (Millipore, Bedford, MA, USA), which were incubated with the appropriate primary antibodies (all at 1:1000 in 1.5% BSA) overnight at 4 °C before incubation with the appropriate HRP-conjugated secondary antibodies (all at 1:6000) for 1 h at room temperature. Immunoreactivity was detected with enhanced chemiluminescence and quantified using Gel-Pro software. The intensities of the target proteins were normalized according to the β–actin or GAPDH content.

### 2.6. Knockdown of Gene Expression

MAPK gene expression knockdown was performed by transfecting small interfering RNAs (siRNAs). The ERK siRNAs (Invitrogen) were AUAUUCUGUCAGGAACCCUGUGUGA and UCACACAGGGUUCCUGACAGAAUAU; the P38 siRNAs (Invitrogen) were UUCAUUCACAGCUAGAUUACUAGGU and ACCUAGUAAUCUAGCUGUGAAUGAA; and the JNK siRNAs (Invitrogen) were AUCUGAAUCACUUGCAAAGAUUUG and CAAAUCUUUGCCAAGUGAUUCAGAU. VSMCs (5 × 10^6^ cells) were transfected with the siRNAs for 48 h using Genjet transfection reagent (SignaGen; Ijamsville, MD, USA). The effects of the siRNAs were evaluated by Western blotting.

### 2.7. Luciferase Promoter Assay

Cells were transfected with p65 luciferase reporter constructs (Promega, Madison, WI, USA). After serum starvation, the transfected cells were pretreated with 10 µM corylin for 1 h; then, the cells were treated with TNF-α for another 6 h. The luciferase activity was performed and detected with a Bright-Glo^tm^ Luciferase Assay System (Promega, Madison, WI, USA) and an Infinite 200 PRO NanoQuant (Tecan Trading AG, Switzerland), respectively.

### 2.8. Immunofluorescence

After fixation in 4% paraformaldehyde, the tissues and cells were incubated with the appropriate primary antibodies (all at 1:100) overnight at 4 °C. Then, the tissues and cells were incubated with the appropriate FITC-conjugated secondary antibody for 1 h at room temperature before counterstaining with diphenyleneiodonium (DPI) and examined under a fluorescence microscope.

### 2.9. Determination of Intracellular Reactive Oxygen Species (ROS) Levels

DHE and DCFH-DA were used to detect the intracellular ROS levels. Briefly, cells were pretreated with corylin, NAC or DPI for 1 h and then coincubated with 10 ng/m TNF-α for 30 min or 3 h and then with 10 μM DHE or 10 μM DCFH-DA for 30 min. The ROS levels were analyzed and observed by fluorescence microscopy.

### 2.10. Cell Cycle Analysis

Cells were grown in 6-well culture plates. The cells were treated with corylin or sirolimus for 1 h and then coincubated with 20 ng/mL PDGF-BB for 24 h. The cells were subsequently trypsinized and stained with PI. Cell cycle progression was assessed with a FACScan cytometer (BD Biosciences, San Jose, CA, USA), and the data were analyzed by ModFit software (v2.0, Verity Software House, Topsham, ME, USA).

### 2.11. Foam Cell Formation and Staining

RAW264.7 cells were pretreated with 20 μM corylin for 1 h and then coincubated with 25 ng/mL oxLDL for 48 h. The cells were stained with 1 μM BODIPY and observed by immunofluorescence microscopy.

### 2.12. Smooth Muscle Cell Wound Injury Repair Assay

After VSMCs were treated with 20 μM corylin or 5 nM sirolimus for 1 h, wounds were inflicted in their monolayers by dragging a sterile pipette tip across the monolayers. A 250-μm cell-free path was created, after which the medium was replaced with fresh medium to remove the cell debris. After being rinsed, the cells were cultured in complete medium containing the vehicle control or 20 ng/mL PDGF-BB with or without 20 μM corylin or 5 nM sirolimus. The degree of cell growth into the wound area was measured after 24 h. Time-lapse images were taken every 10 min after wounding throughout the 24-h period. The cell migration rate was measured after collection of the sequential time-lapse images. Analyses were performed on the sequential phase-contrast images with MetaMorph software (v.7.7 Molecular Devices, CA, USA).

### 2.13. BrdU Incorporation Assay

VSMCs were cultured on gelatin-coated coverslips and serum-starved for 24 h. After pretreatment with 20 μM corylin or 5 nM sirolimus for 1 h, the cells were treated with 20 ng/mL PDGF-BB and 40 mg/mL BrdU for another 24 h. The cells were then fixed with a 95% ethanol/5% acetic acid solution for 30 min and treated with 1 N hydrochloric acid (HCl) for 10 min, after which they were treated with sodium borate to neutralize the HCl. The cells were subsequently incubated with an anti-BrdU antibody or normal IgG antibody overnight at 4 °C before being incubated with FITC-conjugated goat anti-mouse IgG. The cells were then counterstained with 1 µg/mL DAPI and observed under a fluorescence microscope. Cells in six fields were counted under a 20× objective lens to determine the number of BrdU-positive nuclei and the total number of nuclei (DAPI-positive nuclei).

### 2.14. Adhesion Assay

Cultured U937 cells were stained with BCECF-AM. The stained U937 cells were added to the artery or used to treat cells for 60 min at 37 °C. Then, the cells were washed and observed by fluorescence microscopy (Thermo, Wilmington, NC, USA).

### 2.15. MitoTracker staining

VSMCs were cultured on gelatin-coated coverslips and serum-starved for 24 h. After pretreatment with 20 μM corylin or 5 nM sirolimus for 1 h, the cells were treated with 20 ng/mL PDGF-BB for another 24 h. After drug treatment, the cells were incubated with 200 nM MitoTracker (Invitrogen, Carlsbad, CA, USA) for 30 min. The mitochondria morphology of the cells was analyzed and observed by immunofluorescence microscopy (Leica Microsystems; Wetzlar, Germany).

### 2.16. Monocyte Transmigration Assay

HUVEC were grown in transwells. After reaching confluence, cells were pretreated with corylin, NAC, DPI, Parthenolide, VCAM-1 Ab, or IgG Ab for 1 h, then U937 cells were added into upper chamber with or without 10 ng/mL TNF-α treatment and allowed to migrate over 6 h. At the end of the incubation, migrated U937 cells were determined by Coomassive blue staining and count.

### 2.17. Animal Groups and Treatment

Male ApoE-deficient mice (8 w) were purchased from the National Laboratory Animal Center (Taipei, Taiwan) and maintained on a C57/BL6 background. The animal procedures used conformed to the guidelines from Directive 2010/63/EU of the European Parliament for the protection of animals used for scientific purposes. All procedures were performed in accordance with the local institutional guidelines for animal care established by National Taiwan University. The protocol was also approved by the National Taiwan University College of Medicine and College of Public Health’s Institutional Animal Care and Use Committee (IACUC 20180028). The animals were distributed randomly into the following four groups: the animals in group I (control) were fed a standard chow diet (*n* = 12); the animals in group II (cholesterol diet) were fed a high cholesterol diet (0.15% cholesterol; Purina Mills, Inc., Brentwood, MO, USA) for 15 weeks (*n* = 12); the animals in group III (cholesterol diet/corylin, prevention group) were fed a high cholesterol diet plus corylin (50 mg/kg/day) orally for 15 weeks (*n* = 12); and the animals in group IV (cholesterol diet/corylin 7W, treatment group) were fed a high cholesterol diet for 15 weeks and corylin (50 mg/kg/day) orally from weeks 9 to 15 (*n* = 12). After 15 weeks, the mice were euthanized with sodium pentobarbital (120 mg/kg i.p.), and the thoracic aorta was gently removed. Subsequently, the thoracic aorta was fixed in a 4% paraformaldehyde solution for morphological analysis or lysed for Western blot analysis.

### 2.18. Femoral Artery Wire Injury

Male C57BL/6 (8 w) mice were purchased from the National Laboratory Animal Center. This protocol was also approved by the National Taiwan University College of Medicine and College of Public Health’s Institutional Animal Care and Use Committee (IACUC 20150293). All surgical procedures were performed according to the method developed by Sata et al [16]. In brief, endothelial denudation (ED) of the femoral artery was conducted using a spring wire (0.38 mm in diameter, No. C-SF-15-15, COOK, Bloomington, IN, USA). The animals were divided randomly into 2 groups: (1) group I (ED/control) was administered vehicle, and (2) group II (ED/corylin) was administered corylin (50 mg/kg/day). Twenty-eight days after the transluminal mechanical injury, the injured femoral arteries were gently dissected, fixed in 4% paraformaldehyde, Optimal cutting temperature compound (OCT compound)-embedded, and cross-sectioned for morphometric analysis. Every tenth section of the femoral artery was collected and stained with resorcin-fuchsin solution (Sigma, St. Louis, MO, USA) for neointimal formation analysis. The mean neointima/media cross-sectional area (I/M) ratio was calculated using Image-Pro Plus 4.5.

### 2.19. Statistical Analysis

All results were defined as mean ± standard deviation (SD). A single-factor analysis of variance (ANOVA) was used to evaluate the significant differences among the conditions. A two-tailed Student’s *t*-test was used for two group comparisons. A value of *p* < 0.05 was considered statistically significant. All error bars represent the SD.

## 3. Results

### 3.1. Corylin Reduces Inflammation in TNF-α-Treated HUVECs and VSMCs by Downregulating VCAM-1 Expression

**Figure 1:**
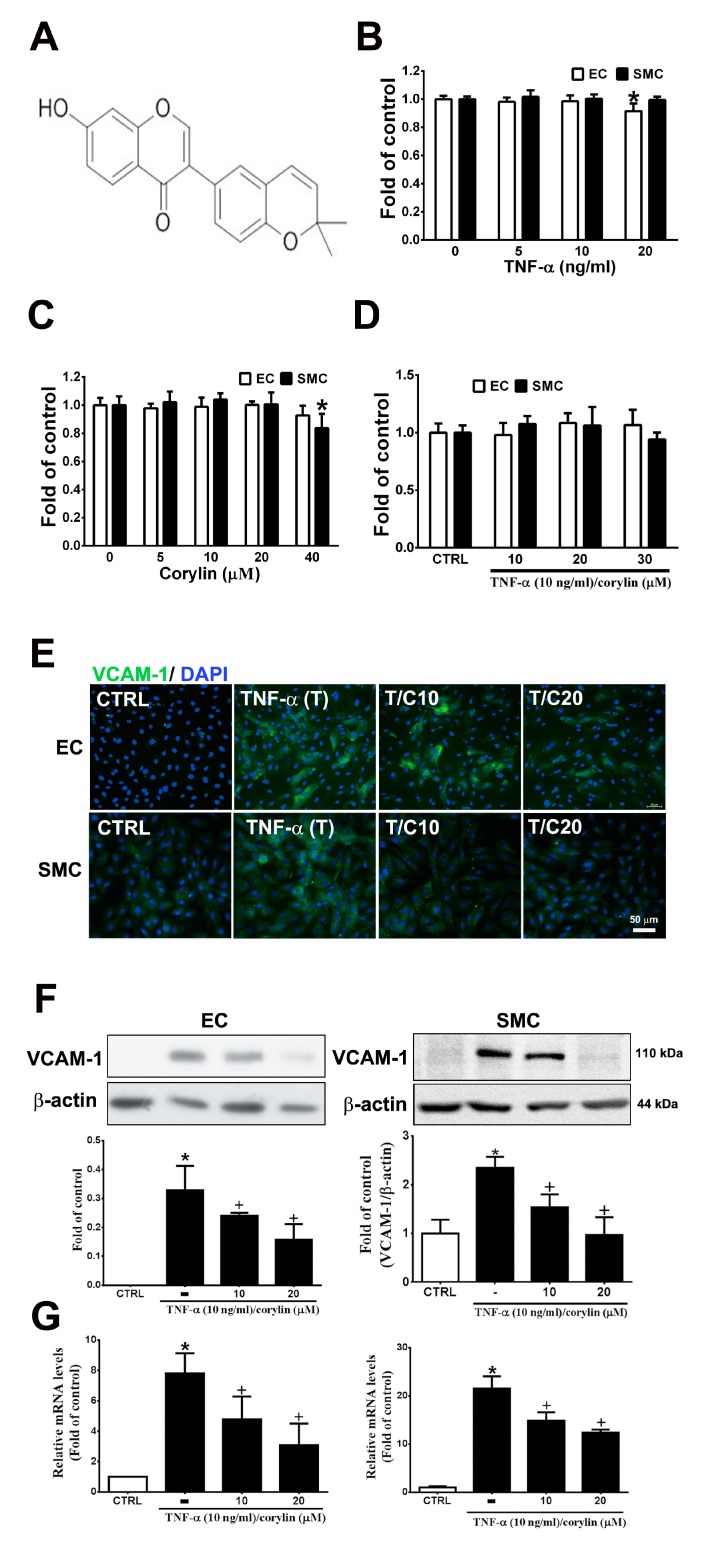
Corylin reduces inflammation in tumor necrosis factor-α (TNF-α)-treated human umbilical vein endothelial cells (HUVECs) and vascular smooth muscle cells (VSMCs) by downregulating vascular cell adhesion protein-1 (VCAM-1) expression. (**A**) Chemical structure of corylin. HUVECs and VSMCs were treated with 0, 5, 10, or 20 ng/mL TNF-α alone for 24 h (**B**), 0, 5, 10, 20, or 40 µM corylin alone for 24 h, (**C**) or pretreated (1 h) with 10, 20 or 30 µM corylin and then treated with 10 ng/mL TNF-α for 24 h. (**D**) SRB assay was performed to observe cell viability. (**E**,**F**) Immunofluorescence staining and Western blot analysis were performed to evaluate the level of VCAM-1 protein expression. β-actin was used as an internal control for sample loading. Nuclei were labeled with DAPI (blue). (**G**) Quantitative real-time PCR was performed to evaluate the VCAM-1 mRNA expression. HUVECs or VSMCs were pretreated (1 h) with 10 or 20 µM corylin (C10 or C20) and then treated with 10 ng/mL TNF-α (T) for 6 h. The data are shown as the mean ± SD. * *p* < 0.05 versus the untreated group (CTRL). ^†^ *p* < 0.05 versus the TNF-α-treated group. Scale bars = 50 μm (**E**).

Inflammation and dysfunction in endothelial cells and VSMCs, which can be induced by TNF-α, play an important role in vascular homeostasis and atherosclerosis progression exacerbation and represent the initial events of atherosclerosis [17,18]. First, we examined the toxicity of TNF-α and corylin (Figure 1A) in HUVECs and VSMCs in culture; during the 24 h culture, 5–40 µM corylin and 5–10 ng/mL TNF-α did not result in cytotoxicity (Figure 1B,C). Similarly, combined TNF-α and corylin treatment did not affect cell viability at these concentrations (Figure 1D). We next investigated the effect of corylin on TNF-α-inducted VCAM-1, ICAM-1 and E-selectin expression in HUVECs and VSMCs. As shown in Figure 1E,F and Figure S1, the immunofluorescence staining and Western blot analysis showed that corylin markedly reduced VCAM-1 expression in TNF-α-treated HUVECs and VSMCs, but not ICAM-1, and E-selectin. Similar data for VCAM-1 mRNA expression were observed in the TNF-α-stimulated HUVECs and VSMCs (Figure 1G). Taken together, these data suggest that corylin treatment results in the inhibition of VCAM-1 protein expression and causes the posttranscriptional modulation of VCAM-1 expression in TNF-α-treated HUVECs and VSMCs.

### 3.2. Corylin Reduces ROS Production in TNF-α-Treated HUVECs and VSMCs

**Figure 2:**
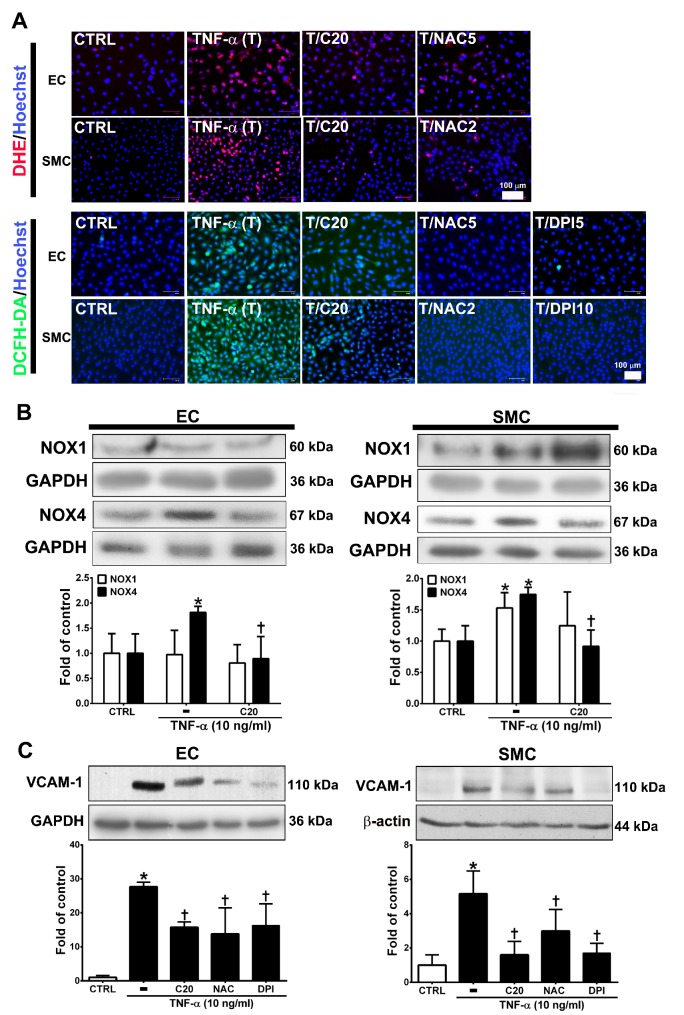
Corylin reduces VCAM-1 expression in TNF-α-treated HUVECs and VSMCs through reactive oxygen species (ROS) inhibition. (**A**) HUVECs and VSMCs were treated with 20 µM corylin (C20), 2 or 5 mM NAC (NAC2 or 5), and 5 or 10 µM DPI (DPI5 or 10) and then treated with 10 ng/mL TNF-α (T) for another 30 min or 3 h. The treated cells were stained with H_2_DCFDA or DHE. The intracellular ROS levels were monitored by fluorescence microscopy. Nuclei were labeled with Hoechst (blue). (**B**) HUVECs and VSMCs were pretreated (1 h) with corylin (C20) and then treated with 10 ng/mL TNF-α (T) for 6 h. Western blot analysis and quantification of NOX1 and NOX4 protein expression. (**C**) HUVECs and VSMCs were treated with 20 µM corylin (C20), 2 or 5 mM NAC, and 5 or 10 µM DPI and then treated with 10 ng/mL TNF-α (T) for 24 h. Western blot analysis for VCAM-1 and quantification of VCAM-1 to GAPDH or β-actin in HUVECs or VSMCs. * *p* < 0.05 versus the untreated group (CTRL). ^†^ *p* < 0.05 versus the TNF-α-treated group. Scale bars = 100 μm (**A**).

ROS are highly associated with inflammatory disease and atherosclerosis progression. [19,20]. Cellular ROS are mainly produced from NADPH oxidases (NOXs) [20,21]. Thus, we evaluated whether the anti-inflammatory role of corylin in TNF-α-treated HUVECs and VSMCs was through ROS regulation. First, we examined the effect of corylin on TNF-α-induced ROS production using DCFH-DA and DHE as probes. The results showed that corylin significantly inhibited hydrogen peroxide and superoxide production in TNF-α-treated HUVECs and VSMCs (Figure 2A). The antioxidant effects were similar to those in the NAC (ROS scavenger) and DPI (NADPH oxidase inhibitor)-treated groups (Figure 2A). Second, we examined whether corylin reduces TNF-α-induced ROS production by reducing NOX4 expression. The results showed that corylin markedly reduced NOX4 expression in TNF-α-treated HUVECs and VSMCs, but not NOX1 (Figure 2B). In addition, the stimulatory effect of TNF-α on VCAM-1 expression was significantly blocked by NAC and DPI treatment in HUVECs and VSMCs (Figure 2C). The results showed that corylin reduced VCAM-1 expression by inhibiting ROS production in TNF-α-treated HUVECs and VSMCs.

### 3.3. Corylin Reduces Monocyte Adhesion and Transmigration in TNF-α-Treated HUVECs by Inhibiting ROS/JNK Signaling

**Figure 3:**
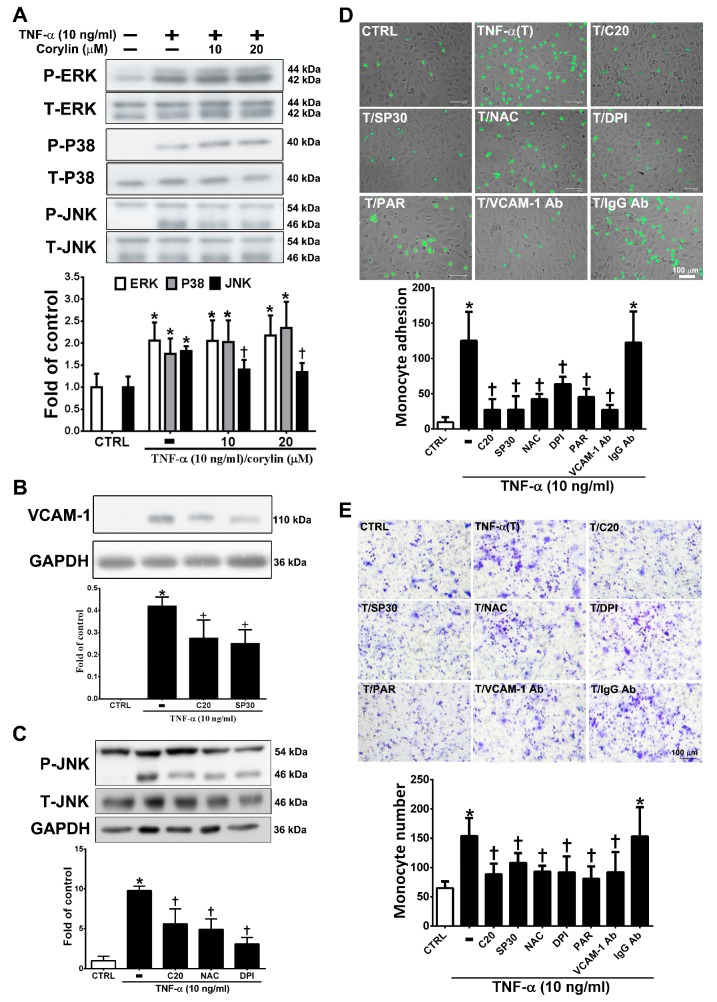
Corylin reduces monocyte adhesion and transmigration in TNF-α-treated HUVECs through inhibiting the ROS/JNK signaling pathway. HUVECs were treated with 20 µM corylin for 1 h and then incubated with or without 10 ng/mL TNF-α for 15 min. Phosphorylated (**A**) ERK, P38, and JNK levels were examined by Immunoblot analysis. Total ERK (T-ERK), total P38 (T-P38), and total JNK (T-JNK) were used as an internal control for sample loading. (**B**) HUVECs were pretreated (1 h) with corylin (20 µM; C20) and a JNK inhibitor (SP600125; 30 nM; SP30) and then treated with 10 ng/mL TNF-α for 24 h. VCAM-1 expression was analyzed by Western blotting analysis. GAPDH was analyzed in parallel as an internal control for protein loading. (**C**) HUVECs were pretreated (1 h) with corylin (20 µM; C20) and antioxidants (NAC; DPI) and then treated with 10 ng/mL TNF-α for 15 min. Phosphorylated JNK expression was analyzed by Western blotting analysis. T-JNK or GAPDH was analyzed in parallel as an internal control for the protein loading. (**D**) Confluent HUVECs were pretreated (1 h) with 20 µM corylin (C20), 30 nM SP600125 (SP30), 5 mM NAC, 5 µM DPI, 10 µM parthenolide (PAR), and 1 µg/ml anti-VCAM-1 antibody or IgG and then with 10 ng/mL TNF-α (T) for 24 h. BCECF/AM-labeled U937 cells were added and cocultured for another 1 h. The adherent U937 cells were counted to evaluate the overall VCAM-1 expression level. (**E**) HUVECs were pretreated (1 h) with 20 µM corylin or 30 nM SP600125 (SP30), 5 mM NAC, 5 µM DPI, 10 µM parthenolide (PAR), and 1 µg/mL anti-VCAM-1 antibody or IgG and then treated with 10 ng/mL TNF-α for 24 h. U937 cells were added to the upper well for another 6 h; then, the transmigrated U937 cells were stained and counted. The data are shown as the mean ± SD. * *p* < 0.05 versus the untreated group (CTRL). ^†^ *p* < 0.05 versus the TNF-α-treated group. Scale bars = 100 μm (**E**).

MAPK activity is well known to strongly affect VCAM-1 expression through TNF-α/ROS axis stimulation [22]. We examined whether corylin inhibits VCAM-1 expression by blocking MAPK pathway activation. As shown in Figure 3A, corylin significantly inhibited c-Jun N-terminal kinase (JNK) phosphorylation but did not inhibit ERK and P38 in TNF-α-treated HUVECs. The observation that pretreatment with SP600125 (a JNK inhibitor) increased corylin’s inhibitory effects on VCAM-1 expression (Figure 3B) further supports this conclusion. In addition, pretreatment with NAC and DPI remarkably repressed JNK signaling activity (Figure 3C). This result showed that corylin inhibited TNF-α-stimulated VCAM-1 expression, likely by inhibiting ROS/JNK signaling. The interaction between monocytes and endothelial cells through VCAM-1 expression induced by TNF-α is important for atherosclerosis development [23,24]. Therefore, monocyte adhesion and transwell assays were performed to observe the effect of corylin on monocyte adhesion and transmigration in TNF-α-treated HUVECs. As shown in Figure 3D, the control confluent HUVECs showed little monocyte adherence, whereas substantially more monocytes bound the TNF-α-treated HUVECs. Corylin pretreatment also significantly reduced the increased binding effect of monocytes to the TNF-α-treated HUVECs. The involvement of VCAM-1 in the adhesion of monocytes to TNF-α-treated HUVECs was examined by pretreating cells with 1 μg/mL anti-VCAM-1 antibody for 1 h before incubation with TNF-α, which resulted in a significant reduction in the binding of monocytes to HUVECs compared to TNF-α-stimulated cells treated with an isotype IgG antibody. These results suggest that VCAM-1 plays a major role in monocyte adhesion to TNF-α-treated HUVECs. The adherence of monocytes to TNF-α-treated HUVECs was also inhibited by 30 nM SP600125 or antioxidant treatment (NAC and DPI) (Figure 3D), and these inhibitory effects did not differ from those observed using the VCAM-1 antibody and corylin. Similar inhibitory effects of corylin were observed in the transmigration assay (Figure 3E). These results showed that corylin inhibits monocyte adherence to TNF-α-stimulated HUVECs and transmigration by inhibiting VCAM-1 expression and that this effect may be mediated by ROS/JNK pathway regulation.

### 3.4. Corylin Reduces VCAM-1 Expression and Monocyte adhesion in TNF-α-Treated VSMCs by Inhibiting ROS/MAP Kinase Activity

**Figure 4:**
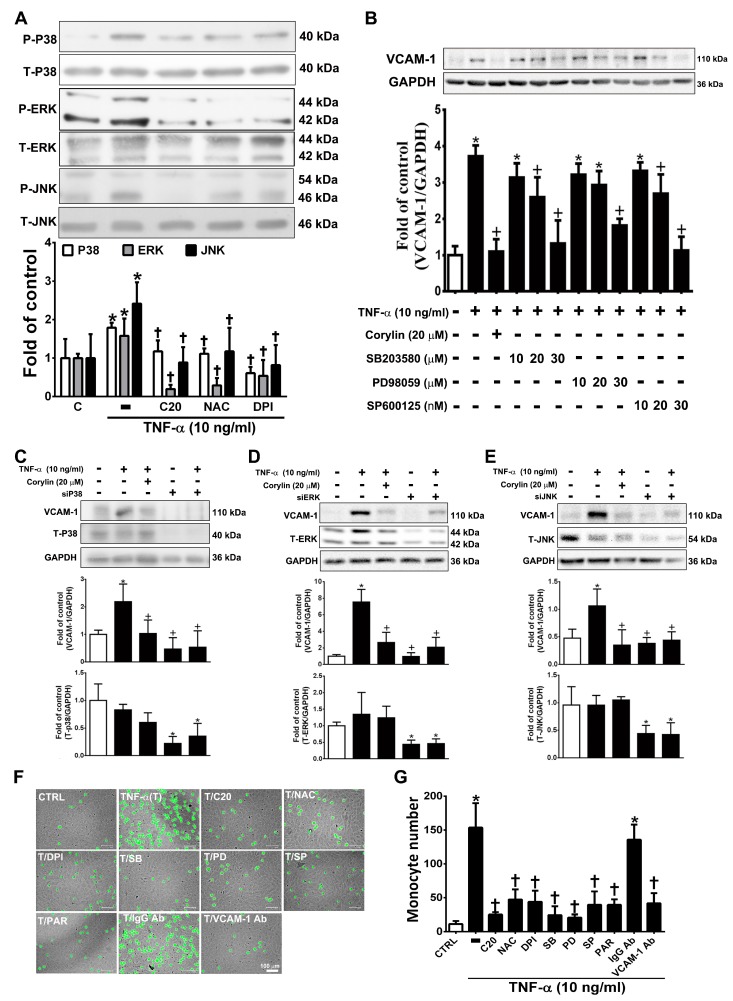
Corylin reduces VCAM-1 expression in TNF-α-treated VSMCs through ROS/MAP kinase inhibition. VSMCs were treated with 20 µM corylin, 2 mM NAC or 10 µM DPI for 1 h and then incubated with or without 10 ng/mL TNF-α for 15 min. (**A**) Phosphorylated P38, ERK, and JNK levels were examined by Immunoblot analysis. Total ERK (T-ERK), total P38 (T-P38), and total JNK (T-JNK) were used as an internal control for sample loading. (**B**) VSMCs were pretreated (1 h) with corylin (20 µM), a P38 inhibitor (SB203580; SB; 10, 20, or 30 µM), PD98059 (PD; 10, 20, or 30 µM), or a JNK inhibitor (SP600125; SP; 10, 20, or 30 nM) and then treated with 10 ng/mL TNF-α for 24 h. Western blot analysis for VCAM-1 and quantification of VCAM-1 to GAPDH in VSMCs. (**C**–**E**) After P38, ERK and JNK silencing, cells were treated with TNF-α and corylin. VCAM-1 expression was determined by Western blot analysis. Total P38 (T-P38), total ERK (T-ERK), and total JNK (T-JNK) proteins were used to examine the siRNA effects. GAPDH was used as an internal control for sample loading. (**F**,**G**) Confluent VSMCs were pretreated (1 h) with 20 µM corylin (C20), 30 µM SB203580 (SB), 30 µM PD98059 (PD), 30 nM SP600125 (SP), 10 µM parthenolide (PAR), and 1 µg/mL anti-VCAM-1 antibody or IgG and then treated with 10 ng/mL TNF-α (T) for 24 h. BCECF/AM-labeled U937 cells were added and cocultured for another 1 h. The adherent U937 cells were counted to evaluate the overall VCAM-1 expression level. The data are shown as the mean ± SD. * *p* < 0.05 versus the untreated group (CTRL). ^†^ *p* < 0.05 versus the TNF-α-treated group. Scale bars = 100 μm (**F**).

Previous studies have shown that MAPKs play an important role in mediating ROS-induced inflammatory processes in VSMCs [25]. Corylin significantly reduced P38, ERK, and JNK phosphorylation in TNF-α-induced VSMCs (Figure 4A). Similar reduction effects were observed in the antioxidant (NAC and DPI)-treated groups (Figure 4A). This result showed that corylin inhibited TNF-α-stimulated P38, ERK, and JNK phosphorylation, likely by inhibiting ROS production. Specific P38, ERK, and JNK inhibitors and MAPK siRNA were used to confirm that corylin exerts anti-inflammatory effects on TNF-α-induced VCAM-1 expression in VSMCs primarily by affecting the MAPK signal transduction pathways. SB302580 (P38 inhibitor), PD98059 (ERK inhibitor), and SP600125 (JNK inhibitor) significantly suppressed the TNF-α-stimulated increases in VCAM-1 expression levels (Figure 4B). Similar effects were observed in the MAPK siRNA experiments (Figure 4C–E). These results indicated that corylin suppressed the TNF-α-induced changes in VCAM-1 expression in VSMCs by inhibiting P38, ERK and JNK activation. Similar data were observed in the monocyte adhesion assays (Figure 4F). The adherence of monocytes to TNF-α-treated VSMCs was also inhibited by PD98059, SB302580, SP600125, or antioxidant (NAC and DPI) treatment (Figure 4F,G), and these inhibitory effects were the same as those induced by treatment with VCAM-1 antibody and corylin. The above studies showed that corylin reduces VCAM-1 expression and monocyte adhesion through the ROS/MAP kinase pathway.

### 3.5. Corylin Reduces NF-κB p65 Activation in TNF-α-Treated HUVECs and VSMCs

**Figure 5:**
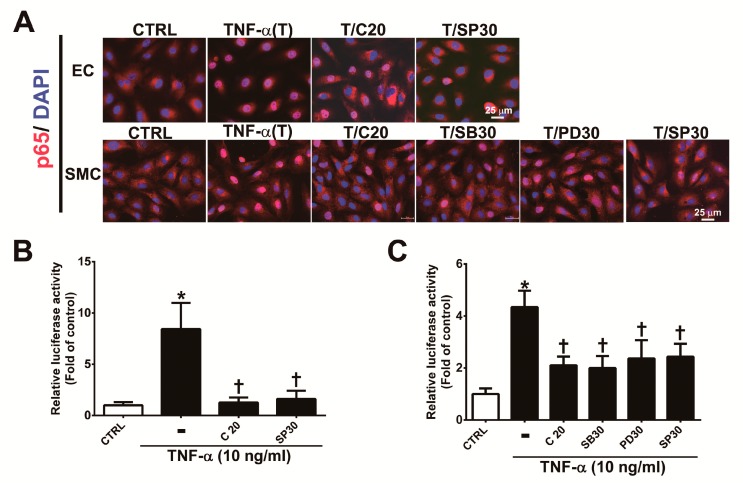
Corylin reduces the activation of NF-κB p65 in TNF-α-treated HUVECs and VSMCs. (**A**) HUVECs were pretreated (1 h) with 20 µM corylin (C20) or 30 nM SP600125 (SP30) and then incubated with 10 ng/mL TNF-α (T) for 15 min. VSMCs were pretreated (1 h) with 20 µM corylin, 30 µM SB203580 (SB30), 30 µM PD98059 (PD30), or 30 nM SP600125 (SP30) and then incubated with 10 ng/mL TNF-α (T) for 15 min. Immunofluorescence staining was performed to examine the nuclear localization and expression level of phosphorylated NF-κB p65 (P-p65). Nuclei were labeled with DAPI (blue). Scale bar = 25 μm. (**B**,**C**) HUVECs and VSMCs were transfected with NF-κB p65 luciferase reporter constructs, pretreated (1 h) with 20 µM corylin (C20), 30 µM SB203580 (SB30), 30 µM PD98059 (PD30), or 30 nM SP600125 (SP30) and then incubated with 10 ng/mL TNF-α for 6 h. Total cell lysates were collected, and luciferase activity was detected. The data are shown as the mean ± SD. * *p* <0.05 versus the untreated group (CTRL). ^†^ *p* < 0.05 versus the TNF-α-treated group.

The MAPK signaling pathway plays an important role in the regulation of the activity of NF-κB p65, which is a transcription factor that regulates VCAM-1 expression [22,25,26]. To determine whether corylin’s antiadhesion effects are partially regulated by the NF-κB pathway, we observed the nuclear translocation and phosphorylation of a p65 subunit of NF-κB by immunofluorescence staining and Western blot analysis. As shown in Figure 5, nuclear NF-κB p65 levels (Figure 5A), phosphorylated p65 subunit expression (Figure S2A,B) and NF-κB p65 activity (Figure 5B,C) were markedly increased in TNF-α-treated HUVECs and VSMCs; however, these effects were attenuated following pretreatment with corylin or SP600125 in HUVECs and corylin, SB302580, PD98059 or SP600125 in VSMCs. In addition, the stimulatory effect of TNF-α on VCAM-1 expression and monocyte adhesion was significantly blocked by parthenolide (NF-kB inhibitor) treatment in HUVECs and VSMCs (Figure S3A,B, Figure 3D,E and Figure 4F,G). Based on these results, we surmised that corylin significantly inhibits TNF-α-induced VCAM-1 expression through the JNK/NF-κB p65 pathway in TNF-α-treated HUVECs and through MAPK/NF-κB p65 in TNF-α-treated VSMCs.

### 3.6. Corylin Inhibited Proliferation and Migration in VSMCs Induced by PDGF-BB through the mTOR Pathway

**Figure 6:**
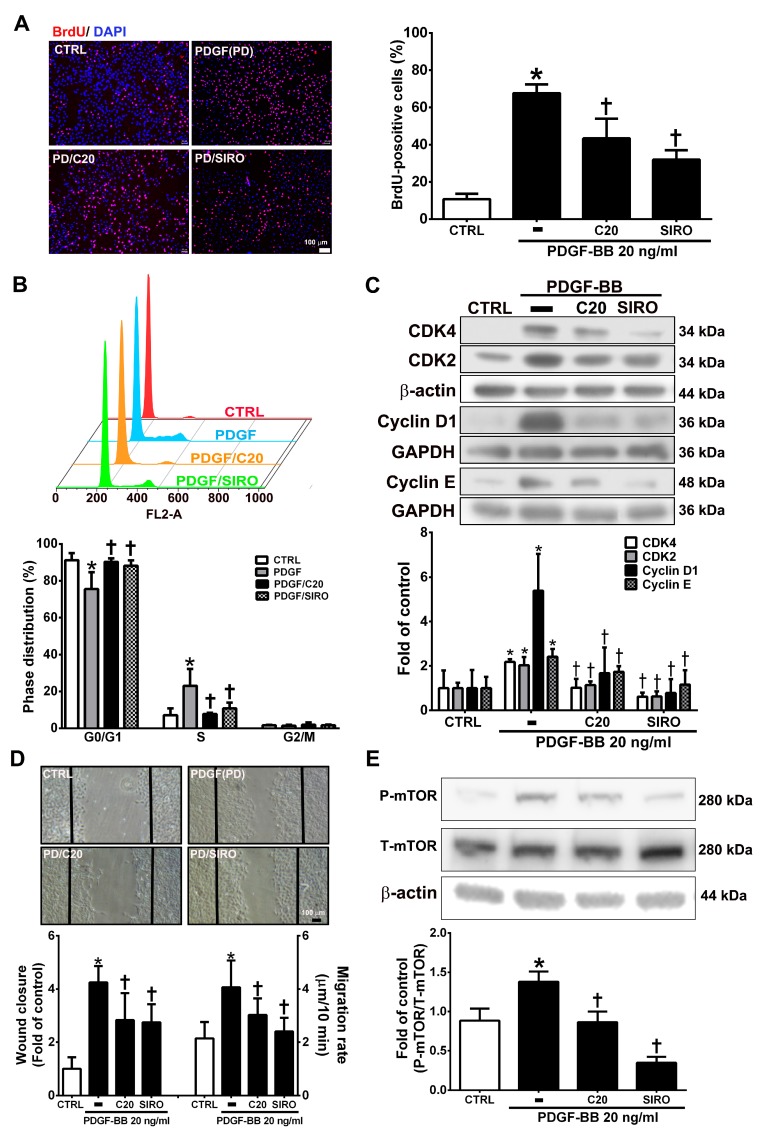
Corylin inhibited proliferation and migration in VSMCs induced by platelet-derived growth factor-BB (PDGF-BB)**.** VSMCs were pretreated (1 h) with 20 µM corylin (C20) or 5 nM sirolimus (SIRO; an mammalian target of rapamycin (mTOR) inhibitor) and then treated with 20 ng/mL PDGF-BB (PD) for 24 h. (**A**) BrdU incorporation, and (**B**) Cell cycle analysis by flow cytometry were used to examine VSMC proliferation. Nuclei were labeled with DAPI (blue). (**C**) Western blot analysis for CDK4, CDK2, Cyclin D1, or Cyclin E and quantification of CDK4, CDK2, Cyclin D1 or Cyclin E to β-actin or GAPDH in VSMCs. (**D**) Wound healing assays was used to measure the VSMCs wound closure and migration rates. Confluent VSMCs were wounded by scratch injury (black lines). (**E**) Western blot analysis for P-mTOR and quantification of P-mTOR to T-mTOR in VSMCs. β-actin was used as an internal control for sample loading. The data are the mean ± SD. * *p* < 0.05 versus the untreated group (CTRL). ^†^ *p* < 0.05 versus the PDGF-BB-treated group. The scale bars = 100 μm.

VSMC proliferation and migration, which can be induced by PDGF-BB, are key factors and events in atherosclerotic lesions and vascular injury [27,28]. The present studies showed that corylin pretreatment remarkably reduced VSMC proliferation and induced G0/G1 phase arrest in PDGF-BB-treated VSMCs (Figure 6A,B). Pretreatment with corylin significantly reduced PDGF-BB-induced CDK4, CDK2, Cyclin D1, and Cyclin E expression (Figure 6C). The wound healing assay was performed to evaluate the antimigratory effects of corylin in PDGF-BB-treated VSMCs. The wound closure and migration rates were remarkably suppressed in corylin-treated group compared with PDGF-BB-treated group (Figure 6D). Previous studies showed that mTOR plays an important role in cell proliferation and migration [29,30]. Therefore, the effects of corylin on mTOR expression were investigated in PDGF-BB-stimulated VSMCs. The phosphorylation of mTOR was upregulated by PDGF-BB treatment, and the increasing effect was significantly reduced by corylin pretreatment (Figure 6E). An mTOR inhibitor (sirolimus) was used to evaluate the effects of corylin on PDGF-BB-induced VSMC proliferation and migration. Sirolimus markedly suppressed PDGF-BB-induced VSMC proliferation and migration (Figure 6A–D). These results showed that the antiproliferative and antimigratory effects of corylin in PDGF-BB-induced VSMCs are regulated by mTOR activation.

### 3.7. Corylin Inhibited Mitochondrial Fission in VSMCs Induced by PDGF-BB through the mTOR/Drp1 Pathway

**Figure 7:**
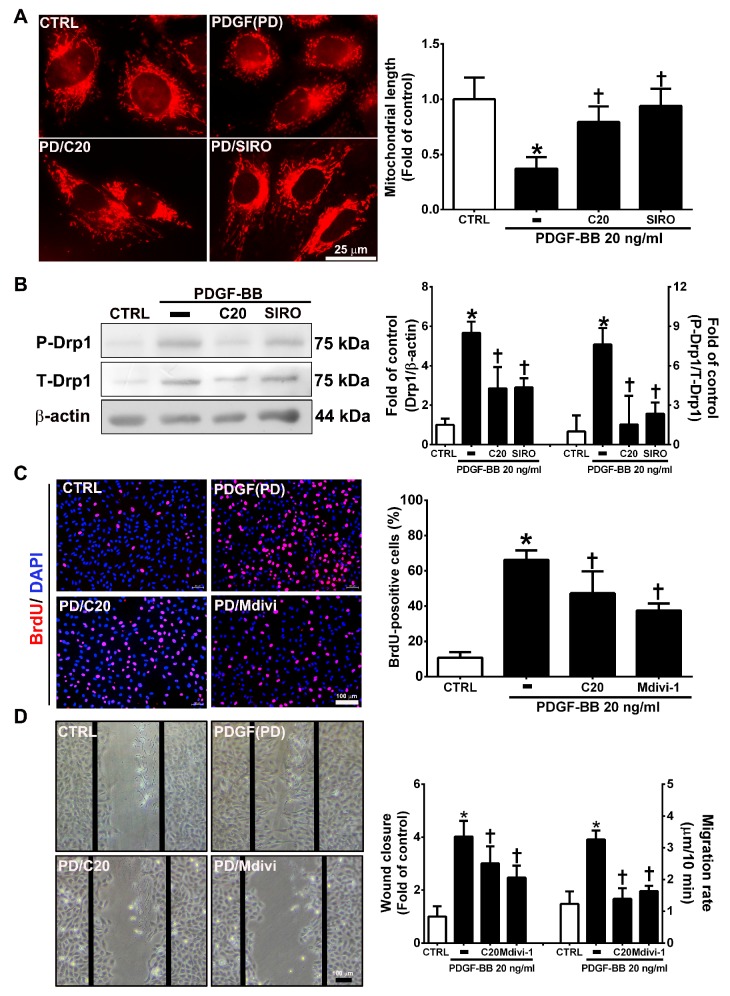
Corylin inhibited mitochondrial fission in VSMCs induced by PDGF-BB through the mammalian target of rapamycin (mTOR)/dynamin-1-like protein 1 (Drp1) pathway. (**A**) Serum-starved VSMCs were pretreated (1 h) with 20 µM corylin (C20) or 5 nM sirolimus (SIRO; an mTOR inhibitor) and then treated with 20 ng/mL PDGF-BB for 6 h. Fluorescence imaging of mitochondrial morphology was observed with MitoTracker dye. Representative photographs are shown in the left panel, and the quantitative data are shown in the right panel. Scale bars = 25 μm. (**B**) Serum-starved VSMCs were pretreated (1 h) with 20 µM corylin or 5 nM sirolimus and then treated with 20 ng/mL PDGF-BB for 24 h. P-Drp1 and T-Drp1 protein expression was determined by Western blot analysis. β-actin was analyzed in parallel as an internal control for protein loading. Serum-starved VSMCs were pretreated (1 h) with 20 µM corylin or 10 µM Mdivi (a specific inhibitor of the mitochondrial fission protein Drp1) and then treated with 20 ng/mL PDGF-BB for 24 h. BrdU incorporation (**C**) and wound healing assays (**D**) were performed to determine VSMC proliferation and migration. Nuclei were labeled with DAPI (blue). The data are the mean±SD. * *p* < 0.05 versus the untreated group (CTRL). ^†^ *p* < 0.05 versus the PDGF-BB-treated group. Scale bars = 100 μm.

Mitochondrial fission plays an essential role in VSMC migration and proliferation regulation [31]. PDGF-BB promotes VSMC migration and proliferation through Drp1-mediated mitochondrial fission [32]. In addition, mTOR controls mitochondrial dynamics and cell survival [33]. To evaluate whether corylin reduces PDGF-BB-induced VSMC proliferation and migration via regulating the mTOR-Drp1 axis, the effects of corylin on Drp1-mediated mitochondrial fission were determined in PDGF-BB-stimulated VSMCs. The results of mitochondrial staining and Western blot analysis showed that corylin significantly reduced PDGF-BB-stimulated mitochondrial fission, Drp1 expression and Drp1 phosphorylation (Figure 7A,B). Similar inhibitory effects were observed in the sirolimus pretreatment group (Figure 7A,B).

Furthermore, the inhibitory effects of PDGF-BB on migration and proliferation were significantly blocked by Mdivi-1 (a specific inhibitor of the mitochondrial fission protein Drp1) pretreatment (Figure 7C,D). The above results suggest that corylin significantly inhibits PDGF-BB-induced proliferation and migration through the mTOR/Drp1 pathway in PDGF-BB-treated VSMCs.

### 3.8. Corylin Reduces Oxidative Stress, VCAM-1 and NOX4 Expression, Monocyte Adhesion, VSMC Proliferation and Atherosclerotic Plaques in Cholesterol Diet-Treated Aortae

**Figure 8:**
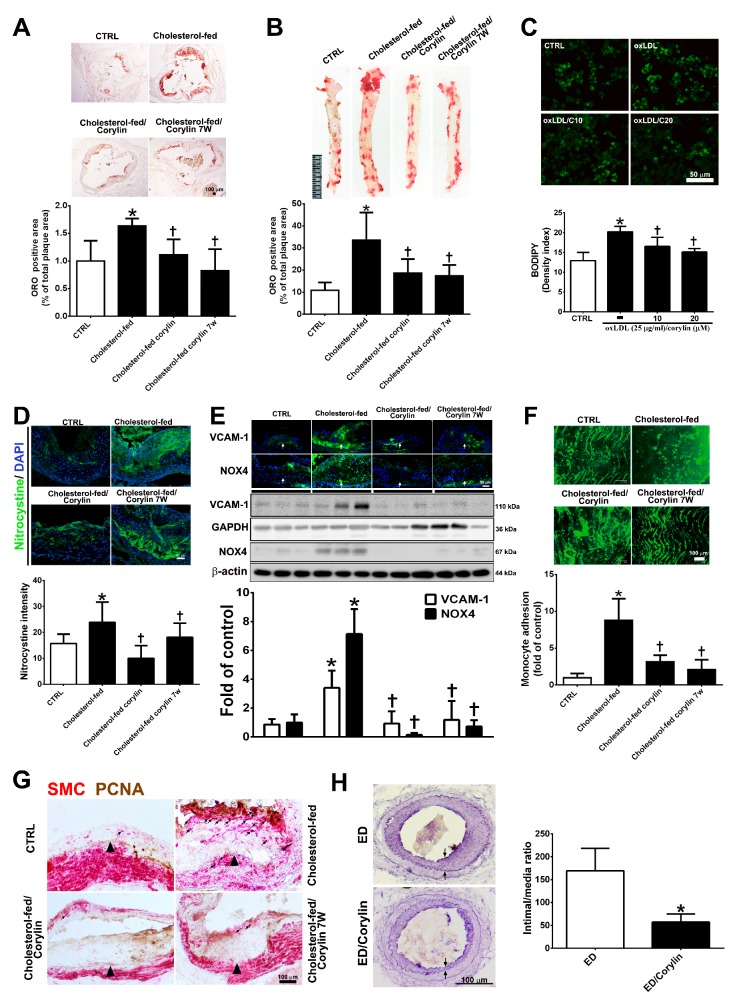
Corylin reduces oxidative stress, VCAM-1 and NOX4 expression, monocyte adhesion, VSMC proliferation and atherosclerotic plaques in high cholesterol diet-treated aortae. (**A**,**B**) Oil Red O staining was performed on aortic sinus and thoracic aortic tissues. Representative photomicrographs are shown with quantifications of the ORO-positive area represented as a percentage of the total atherosclerotic plaque area. (**C**) Lipid droplets were stained using Bodipy. Representative images and quantification of the intensity of lipid staining within the oxLDL-treated RAW264.7 cells. (**D**) Representative images and quantification of the intensity of nitrotyrosine staining within the aortic sinus. Nuclei were labeled with DAPI (blue). (**E**) The expression level of VCAM-1 and NOX-4 in atherosclerotic plaques is shown according to immunohistochemical staining and Western blot analysis of the different groups. Nuclei are labeled with DAPI (blue). GAPDH or β-actin was analyzed in parallel as an internal control for protein loading. The data are expressed as fold changes relative to the control value. (**F**) En face microscopy images illustrate that BCECF/AM-labeled U937 cells bound the endothelium of the thoracic aortae. Quantification of the number of monocytes adhering to the aortae in the different groups is shown. The number represents a percentage of the control. (**G**) Immunohistochemical staining for SMC (red) and PCNA (brown) in cholesterol-fed ApoE mice. The internal elastic lamina is indicated by the arrowheads. The values are provided as the mean ± SD. * *p* < 0.05 versus the CTRL group. ^†^ *p* < 0.05 versus the cholesterol-fed group (CTRL, control group; cholesterol-fed, 0.15% cholesterol-enriched diet was fed for 15 weeks). (**H**) Representative cross-sections of injured femoral arteries. Neointimal hyperplasia is shown between the two arrows. * *p* < 0.05 versus the ED group. Scale bars = 100 μm (**A**,**F**,**G**,**H**), 1 cm (**B**), and 50 μm (**C**,**D**,**E**).

To investigate the anti-atherosclerotic effect of corylin, a histomorphometric analysis of aortic sinus and thoracic aorta sections from ApoE-deficient mice fed a high cholesterol diet for 15 weeks were quantified. As shown in Figure 8A,B, Oil Red O staining of the aortic sinus and thoracic aortae showed that the ratio of the area with atherosclerotic plaques was markedly higher in the high cholesterol diet group than in the standard chow diet group, and corylin treatment (in both the prevention and treatment groups) significantly lessened the atherosclerotic plaque. OxLDL mediates the transformation of macrophages to lipid-laden foam cells, which is a feature of atherosclerotic plaques [34]. In addition, corylin remarkably decreased foam cell formation in oxLDL-treated RAW264.7 macrophages (Figure 8C). Oxidative stress is a key event in atherogenesis [35]. Oxidative stress expression was determined by nitrotyrosine staining. The results showed that oxidative stress marker expression was decreased significantly by corylin treatment (Figure 8D). According to immunohistochemistry staining and Western blot analysis, the VCAM-1 and NOX4 expression levels in the thoracic aortae from ApoE-deficient mice in the cholesterol-fed group were higher than those in the control group (Figure 8E). Corylin treatment (in both the prevention and treatment groups) significantly decreased the VCAM-1 and NOX4 protein expression levels compared with cholesterol feeding alone. Because leukocyte adhesion to the arterial intima plays an important role in the initiation and progression of atherosclerosis, we used labeled monocytes to directly determine whether corylin inhibits early monocyte recruitment to atherosclerotic lesions. The numbers of labeled monocytes in the arteries were higher in high cholesterol diet-fed group than in standard chow diet group. Corylin treatment (in the prevention and treatment groups) dramatically decreased the numbers of monocytes that adhered to the arterial intima compared with cholesterol feeding alone (Figure 8F). The proliferation and migration of VSMCs play an important role in the progression of atherosclerosis [36,37]. To detect VSMC proliferation in atherosclerotic plaques, PCNA and α-actin staining were used. As shown in Figure 8G, the number of PCNA-positive SMCs in the thickened plaques was higher in the cholesterol-fed group than in the corylin-treated group (in the prevention and treatment groups). The antihyperplasia effects of corylin were also observed in denuded mice (Figure 8H). Taken together, these findings show that corylin significantly reduced atherosclerotic lesion progression.

## 4. Discussion

In the present study, our results showed that corylin inhibited TNF-α-stimulated monocyte adhesion to HUVECs and monocyte transmigration by inhibiting VCAM-1 expression partially by suppressing ROS/JNK/NF-κB p65 phosphorylation. In TNF-α-treated VSMCs, corylin suppressed TNF-α-stimulated VCAM-1 expression by inhibiting the ROS/MAPK/NF-κB p65 signaling pathways. In PDGF-BB-treated VSMCs, corylin inhibited VSMC proliferation and migration through the mTOR/Drp1 pathway. Furthermore, corylin treatment significantly reduced the high-cholesterol diet-induced atherosclerotic plaque areas, ROS production, monocyte adherence, and VSMC proliferation in ApoE-deficient mice and inhibited neointimal hyperplasia in endothelial-denuded mice in vivo. These data showed that corylin possesses anti-inflammatory, antioxidative, antiproliferative and antimigratory properties and suggested that corylin might be a candidate therapeutic agent for atherosclerosis.

NOX-derived ROS play a key role in regulating inflammation and adhesion molecule expression in atherosclerotic progression. Previous studies have shown that NOX1 and NOX4, with proinflammatory actions, are implicated in the pathogenesis of cardiovascular disease [38] and are highly associated with atherosclerosis [39,40]. A recent study demonstrated that NOX1 inhibition suppresses VCAM-1 expression in TNF-α-induced VSMCs [41]. In addition, NOX4 inhibition attenuates VCAM-1 induction in HUVECs [42] and VSMCs [43]. The present study showed that corylin significantly reduced ROS production and suppressed VCAM-1 expression in both TNF-α-induced HUVECs and VSMCs. However, corylin reduced only NOX4 expression, not NOX1 expression, in TNF-α-induced HUVECs and VSMCs. The results suggested that the suppression of TNF-α-induced ROS production and VCAM-1 expression by corylin may be regulated by NOX4 inhibition.

The heath and integrity of vascular endothelial cells plays an important role in vascular homeostasis [44,45]. The initiate step of atherosclerotic progression is the activation of endothelial cells, which increase the adhesion molecules expression and then facilitates leukocyte adherence, thereby kicks off the downstream immune response and then results in the atherosclerotic plaque formation. The presence of TNF-α is critical in patients with vascular complications [46,47]. The function of TNF-α is related to increases expression of adhesion molecules VCAM-1, ICAM-1, and E-selectin in the endothelium, where it can also affect endothelial integrity disruption, monocyte adhesion to endothelial cells and monocyte transmigration [48,49,50]. Migrated monocytes can develop into macrophages and lipid-rich foam cells, which have been described to be prominently present in the fibrous caps of advanced atherosclerotic plaques and associated with disease severity [51,52]. Thus, pharmacological agents that block adhesion molecule expression have the potential to inhibit inflammatory diseases, such as atherosclerosis. The study showed that corylin dramatically decreased VCAM-1 expression, monocyte adherence, and monocyte transmigration in TNF-α-treated endothelial cells via inhibiting ROS/JNK activation. Furthermore, our in vivo study also showed that corylin (prevention and treatment groups) remarkably lessened the high-cholesterol diet-induced VCAM-1 expression and atherosclerotic plaque in ApoE-deficient mice in vivo. Taken together, our results suggest that corylin can lessen atherosclerotic plaque through the VCAM-1/ROS/JNK pathway. To the best of our knowledge, this report is the first to show the inhibitory effects of corylin on adhesion molecule expression in endothelial cells.

Numerous studies have demonstrated that VCAM-1 expression in VSMCs facilitates the accumulation of transmigrated leukocytes and the increase in inflammatory reactions within the vascular wall that exacerbate the progression of atherosclerotic plaques [53]. In this study, we evaluated the effects of corylin on the expression of VCAM-1 at both the mRNA and protein levels and observed its effects on monocyte adhesion to VSMCs. These effects were regulated through the ROS/MAPK/NF-κB p65 pathways. The mechanism of corylin’s protective and treatment effects on atherosclerosis progression may involve the inhibition of inflammation and VCAM-1 expression in VSMCs.

The MAPK pathways play regulatory roles in immune response and transcriptional regulation of VCAM-1 expression in TNF-α-induced VSMCs [25,54]. This study showed that corylin pretreatment lessens TNF-α-induced MAPK phosphorylation in VSMCs, including P38, ERK, and JNK. Additionally, the specific inhibition of P38, ERK, and JNK suppressed VCAM-1 protein expression and reduced leukocyte adhesion in TNF-α-induced VSMCs. These results showed that corylin reduces TNF-α-induced VCAM-1 protein expression through multiple pathways. In addition, corylin inhibits TNF-α-induced VCAM-1 protein expression in HUVECs, and the inhibitory effect of corylin in TNF-α-stimulated HUVECs is achieved mainly via JNK phosphorylation inhibition rather than through ERK and P38 activity. The different regulatory pathways related to vascular cells affected by corylin may have resulted from the differences in the cell types investigated and differences in culture conditions. Taken together, the present data offer a possible mechanism underlying the inhibitory effects of corylin and imply that corylin has potential for the prevention or treatment of atherosclerotic cardiovascular disease.

Mitochondrial dynamics are involved in physiological function and pathological conditions such as cell proliferation and migration [55]. Mitochondrial dynamics, including fusion and fission, are tightly regulated by dynamin-related GTPase Drp1, mitofusins (Mfn1 and Mfn2) and optic atrophy 1 (OPA1) [56]. Previous studies have shown that PDGF-BB treatment enhanced Drp1-mediated mitochondria fission [31,32,57], and an increasing number of studies have shown that Drp1-mediated mitochondria fission was regulated by SIRT1, MUC, CaMKII and PKC [58,59,60]. Recently, it was also shown that mTOR signal inhibition suppressed mitochondria fission [61]. According to the above studies, the dynamic regulation of mitochondria occurs through multiple signaling pathways. In the present study, we showed that corylin decreases PDGF-BB-induced VSMC proliferation and migration through mitochondria fission inhibition by an mTOR/Drp1 signaling pathway.

## 5. Conclusions

In summary, the results of the present study demonstrate that corylin can inhibit VCAM-1 expression in HUVECs and VSMCs. The inhibition of the expression of cell adhesion molecules inhibited monocyte adhesion to TNF-α-stimulated HUVECs and VSMCs, demonstrating that corylin has pharmacological activity in vascular cells. The action of corylin resulted from VCAM-1 expression suppression, ROS/JNK pathway activation and NF-κB p65 activation inhibition in TNF-α-stimulated HUVECs. In TNF-α-stimulated VSMCs, corylin decreased VCAM-1 expression via ROS/MAPK phosphorylation and NF-κB p65 activation inhibition. In PDGF-BB-treated VSMCs, corylin inhibited VSMC proliferation and migration through the mTOR/Drp1 pathway. In addition, dietary supplementation with corylin reduced VCAM-1 expression, monocyte adhesion in aortae, ROS production, VSMC proliferation, and the atherosclerotic plaque area in high-cholesterol diet-induced ApoE-deficient mice. In addition, corylin prevented VSMC proliferation and intimal hyperplasia in denudated-femoral arteries. These findings provide evidence that corylin may be a novel agent that could protect the vasculature from TNF-α-induced inflammation and dysfunction and PDGF-BB-induced proliferation and migration.

## Supplementary Materials

The following are available online at https://www.mdpi.com/2076-3921/9/4/275/s1, Figure S1: Corylin has no effect on ICAM-1 and E-selectin expression in TNF-α-treated HUVECs and VSMCs. HUVECs (A) and serum-starved VSMCs (B) were pretreated (1 h) with corylin (10 or 20 µM) and then treated with 10 ng/mL TNF-α for 24 h. ICAM-1 and E-selectin expression were analyzed by western blotting. β-actin was used as an internal control for sample loading. CTRL, control group, VSMCs were cultured without any treatment. The data are provided as the mean ± SD. * *p* < 0.05, Figure S2: Corylin reduces the activation of NF-κB p65 in TNF-α-treated HUVECs and VSMCs. (A,B)HUVECs were pretreated (1 h) with 20 µM corylin or 30 nM SP600125 (SP30) and then incubated with 10 ng/mL TNF-α (T) for 15 min. VSMCs were pretreated (1 h) with 20 µM corylin, 30 µM SB203580 (SB30), 30 µM PD98059 (PD30), or 30 nM SP600125 (SP30) and then incubated with 10 ng/mL TNF-α (T) for 15 min. Western blot analysis for phosphorylated NF-κB p65 (P-p65) and quantification of P-p65 to Total NF-κB p65 (T-p65) in VSMCs. The data are provided as the mean ± SD. * *p* < 0.05 versus the untreated group (CTRL). ^†^ *p* < 0.05 versus the TNF-α-treated group, Figure S3: Corylin reduces the VCAM-1 expression in TNF-α-treated HUVECs and VSMCs via NF-κB p65 pathway. (A,B) HUVECs and VSMCs were pretreated (1 h) with corylin (20 µM; C20) or 10 µM parthenolide (PAR) and then treated with 10 ng/mL TNF-α for 24 h. Western blot analysis for VCAM-1 and quantification of VCAM-1 to GAPDH in VSMCs. The data are provided as the mean ± SD. * *p* < 0.05 versus the untreated group (CTRL). ^†^ *p* < 0.05 versus the TNF-α-treated group.
