# Supplementary material for: Corylin Inhibits Vascular Cell Inflammation, Proliferation and Migration and Reduces Atherosclerosis in ApoE-Deficient Mice
Source: Antioxidants (Basel). 2020 Mar 25;9(4):275. doi: 10.3390/antiox9040275 (PMC7222202; doi:10.3390/antiox9040275)
Supplement: Supplementary file 1 [file antioxidants-09-00275-s001.pdf]

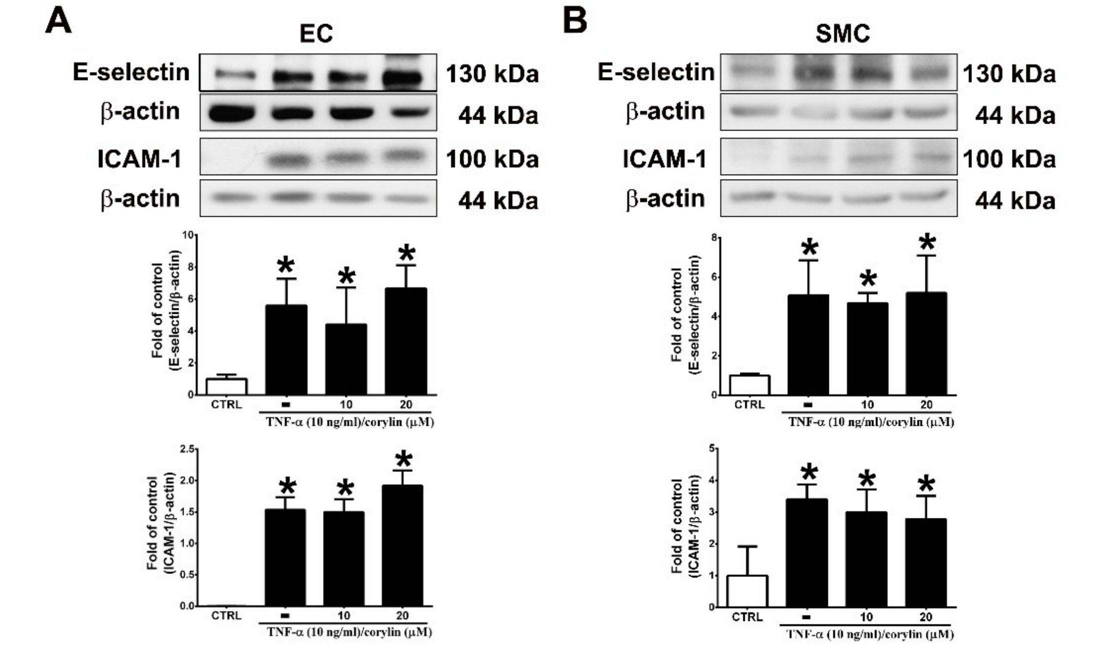

Figure S1. Corylin has no effect on ICAM-1 and E-selectin expression in TNF-α-treated HUVECs and VSMCs. HUVECs (**A**) and serum-starved VSMCs (**B**) were pretreated (1 h) with corylin (10 or 20 μM) and then treated with 10 ng/mL TNF-α for 24 h. ICAM-1 and E-selectin expression were analyzed by western blotting. β-actin was used as an internal control for sample loading. CTRL, control group, VSMCs were cultured without any treatment. The data are provided as the mean ± SD. * $p < 0.05$.

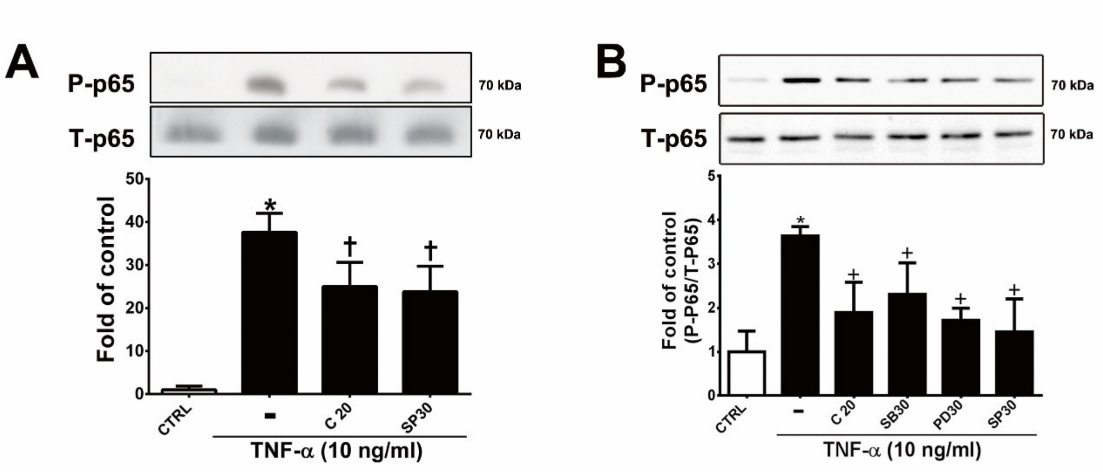

Figure S2. Corylin reduces the activation of NF-κB p65 in TNF-α-treated HUVECs and VSMCs. (**A**,**B**)HUVECs were pretreated (1 h) with 20 μM corylin or 30 nM SP600125 (SP30) and then incubated with 10 ng/mL TNF-α (T) for 15 min. VSMCs were pretreated (1 h) with 20 μM corylin, 30 μM SB203580 (SB30), 30 μM PD98059 (PD30), or 30 nM SP600125 (SP30) and then incubated with 10 ng/mL TNF-α (T) for 15 min. Western blot analysis for phosphorylated NF-κB p65 (P-p65) and quantification of P-p65 to Total NF-κB p65 (T-p65) in VSMCs. The data are provided as the mean ± SD. * $p < 0.05$ versus the untreated group (CTRL). † $p < 0.05$ versus the TNF-α-treated group.

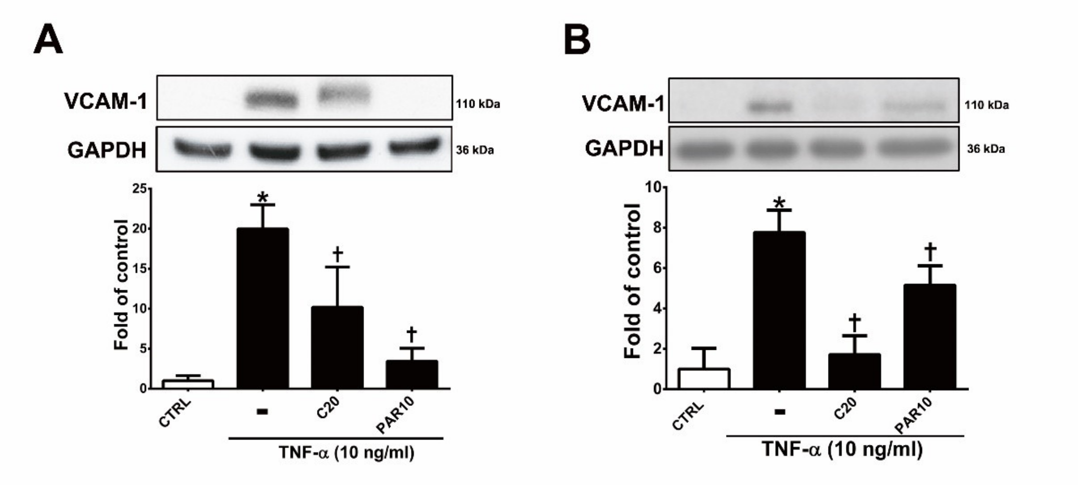

Figure S3. Corylin reduces the VCAM-1 expression in TNF-α-treated HUVECs and VSMCs via NF-κB p65 pathway. (**A**,**B**) HUVECs and VSMCs were pretreated (1 h) with corylin (20 μM; C20) or 10 μM parthenolide (PAR) and then treated with 10 ng/mL TNF-α for 24 h. Western blot analysis for VCAM-1 and quantification of VCAM-1 to GAPDH in VSMCs. The data are provided as the mean ± SD. * $p < 0.05$ versus the untreated group (CTRL). † $p < 0.05$ versus the TNF-α-treated group.
